# Degradation of folic acid wastewater by electro-Fenton with three-dimensional electrode and its kinetic study

**DOI:** 10.1098/rsos.170926

**Published:** 2018-01-17

**Authors:** Gu Xiaochao, Lu Xuebin, Tian Jin, Li Xiaoyun, Zhou Bin, Zheng Xujing, Xu Jin

**Affiliations:** 1 Tianjin Key Laboratory of Indoor Air Environmental Quality Control, School of Environmental Science and Engineering, Tianjin University, Tianjin 300072, People's Republic of China; 2 School of Management, Tianjin University of Technology, Tianjin 300384, People's Republic of China; 3 The Administrative Center for China's Agenda 21, Beijing 100038, People's Republic of China

**Keywords:** folic acid wastewater, electro-Fenton, three-dimensional electrode, kinetic analysis

## Abstract

The three-dimensional electro-Fenton method was used in the folic acid wastewater pretreatment process. In this study, we researched the degradation of folic acid and the effects of different parameters such as the air sparging rate, current density, pH and reaction time on chemical oxygen demand (COD) removal in folic acid wastewater. A four-level and four-factor orthogonal test was designed and optimal reaction conditions to pretreat folic acid wastewater by three-dimensional electrode were determined: air sparge rate 0.75 l min^−1^, current density 10.26 mA cm^−2^, pH 5 and reaction time 90 min. Under these conditions, the removal of COD reached 94.87%. LC-MS results showed that the electro-Fenton method led to an initial folic acid decomposition into *p*-aminobenzoyl-glutamic acid (PGA) and xanthopterin (XA); then part of the XA was oxidized to pterine-6-carboxylic acid (PCA) and the remaining part of XA was converted to pterin and carbon dioxide. The kinetics analysis of the folic acid degradation process during pretreatment was carried out by using simulated folic acid wastewater, and it could be proved that the degradation of folic acid by using the three-dimensional electro-Fenton method was a second-order reaction process. This study provided a reference for industrial folic acid treatment.

## 1. Introduction

Folic acid, which plays a crucial rule in several important biological processes in living cell systems, is a member of the water-soluble B-complex vitamins family [1]. Folic acid wastewater is one of the most polluting effluents produced by pharmaceutical processing [2], which is characterized by a high chemical oxygen demand (COD), high salinity, high chroma and poor biodegradability. The arbitrary discharge of sewage not only leads to significant environmental problems, but also seriously affects water quality and drinking water supplies and may constitute a potential risk to ecosystems and human and animal welfare in the long term. To solve the environmental problems, effective and environmentally friendly technologies need to be employed to treat this kind of wastewater [3].

The Fenton oxidation method is mainly used for the degradation of poorly biodegradable organic compounds by the reaction of ferrous salts and hydrogen peroxide [4]. Under acidic conditions, the hydrogen peroxide is decomposed to produce ·OH catalysed by Fe^2+^. The organic radicals generated in the reaction can continue to participate in the chain reaction of ·OH, or after the formation of organic peroxides, with further oxidative decomposition reaction until the final products of CO_2_ and H_2_O, so as to achieve the purpose of degradation of organic matter [5,6]. Martinez *et al*. [7] performed on experiment on pharmaceutical wastewater treatment by the Fenton oxidation method. When the COD of wastewater was 36 200 mg l^−1^, biochemical oxygen demand (BOD_5_)/COD = 0.08, H_2_O_2_ was 3 mg l^−1^, Fe^2+^ was 0.3 mg l^−1^ and the maximum removal of COD was 56.4%. Thus the Fenton oxidation method is suitable for sewage, and the required time is short. However, because of the high cost, and large amounts of ferric hydroxide and ferrous hydroxide precipitation causing secondary pollution problems, it still needs to be improved [8].

Electro-Fenton research began in the 1980s [8], mainly used for the treatment of difficult degradation of organic compounds, such as pesticides, dyes and other compounds [8–14]. This technique relies mainly on the production of highly active groups, ·OH, to rapidly mineralize contaminants or to improve their biodegradability. Compared with the traditional method, the electro-Fenton oxidation method has the following three advantages: (i) during the traditional Fenton's oxidation, the main agent of hydrogen peroxide must be added, while it can be automatically generated in the electro-Fenton reaction, which avoids the risks of transportation and storage, and at the same time with relative savings in cost [15]; (ii) organic matter oxidation is thorough, the treatment effect is good and the energy consumption is relatively low [16]; and (iii) the parameters that need to be controlled are only voltage and current, for which it is easy to realize automatic control [17,18].

In the reaction system, under the acidic condition, the cathode oxygen reacts with the hydrogen ion, and the reaction generates H_2_O_2_, as shown in the following [18–24]:

1.1 $${\text{O}}_{2}+2{\text{H}}^{+}+2{\text{e}}^{-}\to {\text{H}}_{2}{\text{O}}_{2}.$$

The H_2_O_2_ and the addition of Fe^2+^ reacts to generate ·OH and a series of chain reactions:

1.2 $${\text{H}}_{2}{\text{O}}_{2}+{\text{Fe}}^{2+}\to {\text{Fe}}^{3+}+\cdot \text{OH}+{\text{OH}}^{-}$$

and

1.3 $$\text{RH}+\cdot \text{OH}\to \cdot \text{R}+{\text{H}}_{2}\text{O}.$$

Then Fe^3+^ regenerates Fe^2+^, and this process also effectively reduces the production of iron mud [17–20].

1.4 $$\begin{array}{r} \text{F}{\text{e}}^{3+}+{\text{H}}_{2}{\text{O}}_{2}\to \text{F}{\text{e}}^{2+}+\cdot \text{H}{\text{O}}_{2}+{\text{H}}^{+}, \end{array}$$

1.5 $$\begin{array}{r} \text{F}{\text{e}}^{3+}+\cdot \text{H}{\text{O}}_{2}\to \text{F}{\text{e}}^{2+}+{\text{O}}_{2}+{\text{H}}^{+}, \end{array}$$

1.6 $$\begin{array}{r} \text{F}{\text{e}}^{3+}+{\text{e}}^{-}\to \text{F}{\text{e}}^{2+} \end{array}$$

1.7 $$\begin{array}{r} \;\text{and}\;\text{F}{\text{e}}^{3+}+\cdot \text{R}\to {\text{R}}^{+}+\text{F}{{\text{e}}^{2}}^{+}. \end{array}$$

With a wide range of applications, fast reaction rate and strong oxidation ability, it has been widely used and has shown a great advantage in the treatment of pharmaceutical wastewater. Pharmaceutical wastewater is one of the most difficult-to-treat organic wastewaters with high concentrations of contaminants to deal with. It is characterized by complex composition, high concentration of organic pollutants, high values and volatility of COD and BOD_5_, high chroma, deep toxicity and high suspended substance concentration, with the BOD_5_/COD values being quite different [25–27]. In 2016, Tian *et al*. [28] studied the process of high-efficiency degradation of rhodamine B using the electro-Fenton method to modify the graphite felt gas diffusion electrode. They used modified graphite felt after the gas diffusion electrode as cathode material with the cathode current density at 50 A m^−2^, with 0.05 M Na_2_SO_4_, air sparge rate 1.0 l min^−1^ and a reaction time of 20 min, which caused a rhodamine B removal rate of 98.49%. In 2015, Zhang *et al*. [19] developed an innovative bioelectro-Fenton system capable of alternate switching between the microbial electrolysis cell and microbial fuel cell mode of operation, which could supply H_2_O_2_ and remove residual H_2_O_2_. Liu *et al*. [29] found that N-doped graphite felt showed a significant improvement in degradation of levofloxacin, and total organic carbon removal could reach 92% at 200 mA for 480 min, which was higher than with the unmodified one at twice the current. It can be seen that the application of the electro-Fenton oxidation method could be suitable for the treatment of folic acid wastewater.

In recent years, scholars have conducted more research on the three-dimensional electrode. The three-dimensional electrode is based on the traditional two-dimensional electrode by filling with granular or other clastic working electrode material as the third pole. The three-dimensional electrode has a larger electrode surface, higher mass transfer, high current efficiency and good treatment effect [30]. Fockedey [31] used the three-dimensional electrode to treat organic wastewater containing phenol with a Sb/SO_2_ titanium foam anode and reticulated vitreous carbon cathode. The system was affected by the operating conditions, and the energy consumption was 5 kWh kg^−1^ COD. Polcaro [32] had shown that with carbon bead as the anode, chlorophenols can be completely oxidized, and no intermediate products are generated. In the treatment of organic wastewater, the three-dimensional electrode shows a good application prospect, but due to lack of in-depth research, further study is required on the mechanism, especially the actual reaction process on the electrode surface, reaction thermodynamics, kinetics and practical applications. In addition, powdered activated carbon (PAC) is a good adsorption conductive material which can be used as an electrode, but in the application of the three-dimensional electrode has not been used extensively. Loubna *et al.* [33] found that the degradation of m-cresol by the electro-Fenton method with iron-loaded activated carbon is mainly through adsorption and oxidation. However, we hope that the activated carbon can be used as the third pole material to form a three-dimensional electrode. Therefore, activated carbon is used after saturated folic acid wastewater.

In this work, a novel three-dimensional electro-Fenton method is used to remove COD from folic acid wastewater, and the mechanism of folic acid degradation was studied. We are concerned about the efficiency of the three-dimensional PAC cathode and the influences of several operating parameters on COD removal from the folic acid wastewater. The effects of the air sparging rate, current density and reaction time on the effect of COD removal were evaluated; at the same time, we analysed in detail the degradation products of folic acid by LC-MS. Finally, the kinetics of the reaction of the degradation of folic acid were proposed.

## 2. Material and methods

### 2.1. Folic acid wastewater and powdered activated carbon

Experimental raw water was obtained from a folic acid production plant in Hebei Province, with COD = 1041.15 mg l^−1^. Pure folic acid used in the experimental analysis of kinetics was purchased from Tianjin Kermel Chemical Reagent Co. Ltd. PAC is washed with distilled water before use, and placed in the oven and baked to a constant weight at 50°C.

PAC has been used more and more in water treatment because of its large specific surface and strong adsorptive characteristic. It should be stated that all the graphite felt and activated carbon are absorbed by folic acid wastewater, and PAC was saturated and eliminated the effect of adsorption. Normally, PAC is used as the adsorbent to remove COD in wastewater, and it needs to be regenerated, which incurs a high cost. Characteristics of PAC are given in the electronic supplementary material, table S1.

### 2.2. Experimental apparatus

**Figure 1.**
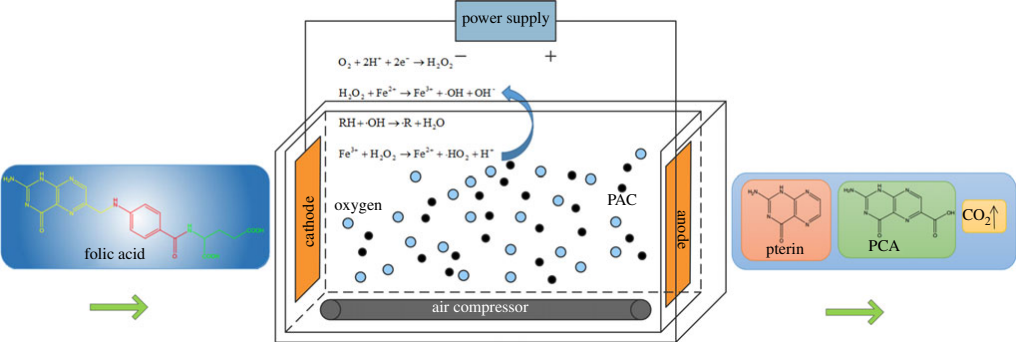
Experimental apparatus.

The material of the electro-chemical reactor is made of organic glass with a size of 200 mm × 40 mm ×80 mm (L × W × H), as shown in figure 1. The anode is titanium plate coated with ruthenium–iridium and the cathode is a titanium net covered with activated carbon fibre. The size of both anode and cathode is 150 mm × 85 mm × 1.5 mm (L × W × T). We cut the activated carbon fibre to the same size as the cathode plate and wrapped it on the cathode plate. PAC is directly added to the reactor as a third pole. The power supply is a DH1716A-10-type stabilized voltage with stabilized flow control power supply. The bottom of the reactor is provided with a microporous aeration strip which is connected to an air compressor to enhance the mass transfer function of the wastewater in the reactor. A micro-agitator is arranged in the reactor to ensure the distribution of the PAC in the reactor and improve the reaction result.

### 2.3. Electro-Fenton process and analytical method

Folic acid wastewater (150 ml) was treated by electro-Fenton processes for analysis; 20% (v/v) of H_2_SO_4_ and 2 mol l^−1^ NaOH were prepared to adjust the pH to the desired value. The power supply was turned on after adding the desired amount of 1.5 g l^−1^ PAC, and a certain amount of FeSO_4_ to maintain a certain concentration of ferrous ions. At the same time, the system was aerated and stirred. The concentration of Fe^2+^ was determined according to the best concentration given in the literature [34].

The oxidation reaction was stopped after a period. The sample needs to be static to remove hydrogen peroxide. The pH was adjusted to 10 by adding a certain amount of 2 mol l^−1^ NaOH after sampling. The samples were filtered after 8000 r min^−1^ centrifugation to remove precipitates (including PAC and Fe^2+^) before COD analysis. The method of COD determination was done with reference to the standard of HJ 828–2017 in China [35]. Each sample was analysed three times repeatedly. We took the average values of the results, which are shown in the figures in the following section.

### 2.4. LC-MS analytical method

The folic acid was analysed by UV spectrophotometry (L5S). Concentrations of folic acid in adsorption experiments were determined at a wavelength of 281 nm at a pH of 6.8.

HPLC analyses were carried out and the column used for the chromatography was a C-18 column (4.6 mm × 250 mm, 5 µm) at a flow rate of 1.0 ml min^−1^ with a column temperature of 25°C and injection volume of 20 µl. The HPLC mobile phases consisted of ammonium acetate (including 0.1% aqueous formic acid) in methanol.

The mass spectra were detected by multiple ion reaction monitoring (MRM), with a post-column separation ratio of 1 : 3. The spectrometer was operated in the full scan mode (100–600 m z^−1^) using positive electrospray ionization as the main mass spectrometry condition for analysis of folic acid.

### 2.5 Orthogonal test

The orthogonal design is a method of multiple factors and multiple levels; it is based on the orthogonality selected from comprehensive tests of some representative point tests; orthogonal test design is a method of fractional factorial design [36]. The experimental set-up is shown in the electronic supplementary material.

## 3. Results and discussion

### 3.1. Effect of powdered activated carbon on the electro-Fenton oxidation of folic acid wastewater

To study the degradation effect of PAC as an electrode for folic acid wastewater, the adsorptive effect of PAC must eliminate COD. The COD of the initial folic acid wastewater is over 1100 mg l^−1^. When PAC was added and reached the adsorption saturation, the organic compound was adsorbed by PAC and the COD dropped noticeably to 499.40 mg l^−1^; the removal of COD reached 52.02%. It can be concluded that the removal of COD can reach 50.02% when PAC is used as the adsorbent, that is to say, we must consider the adsorption by PAC in the experimental process; while studying the effect of electro-Fenton, we should consider the adsorptive effect of PAC. When the current density was fixed at 2.46 mA cm^−2^ with PAC as the electrode, the COD showed a sharp decrease and reached 71.2 mg l^−1^; the COD was reduced by 93.16%. This result indicated that apart from absorption, the removal of COD could reach 43.14% when PAC was used as electrode.

**Figure 2.**
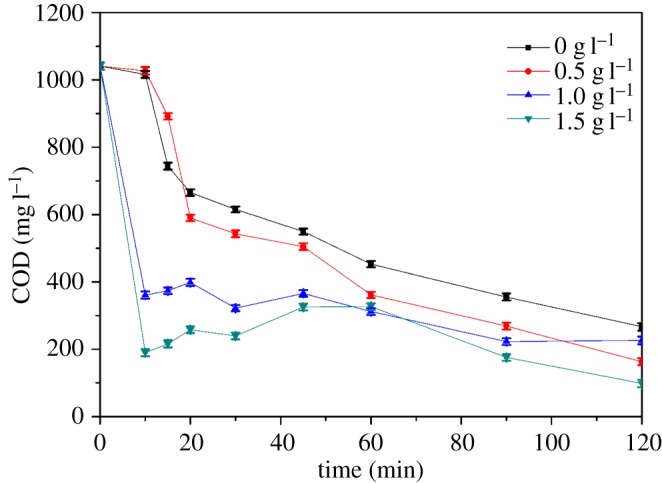
Effects of different PAC dosage on COD removal. Conditions: initial pH 3; air sparging rate 0.5 l min^−1^, [Fe^2+^] 1.0 mM, PAC 1.5 g l^−1^, current density 2.64 mA cm^−2^.

The dosage of PAC in the three-dimensional electrode in this work is determined to be 1.5 g l^−1^ according to figure 2. When the PAC dosage is 0 g l^−1^ and 0.5 g l^−1^, the COD in the solution decreases gradually with the increase in time. When the dosage of PAC was 1 g l^−1^ and 1.5 g l^−1^, the COD fluctuations eventually decreased with the increase in time, which may be due to increased additional dosage of PAC causing increased electrical conductivity. The experimental results showed that when the PAC dosage is 1.5 g l^−1^, removal of organic matter in wastewater is the most efficient; if the dosage of PAC were to continue to increase, it may lead to short circuit of the battery, thereby reducing the current efficiency.

### 3.2 Effect of pH, current density, aeration rate and reaction time on folic acid wastewater pretreatment

To understand the influence of various factors such as pH, current density, air sparge rate and reaction time on the treatment effect of electro-Fenton oxidation of folic acid and optimized experimental condition, 16 sets of orthogonal tests with four factors and four levels were designed [36]; the results and analysis are given in table 1.

**Table 1.** Experimental results of sixteen groups of orthogonal experiments. Notes: The *K* is the average of a certain factor and level, and can be used to determine the global optimal extraction condition; and *R* is the range of *K*, which can show the effect of a certain factor on the removal efficiency.

experiment	pH	current density (mA cm^−2^)	oxygen sparging rate (l min^−1^)	reaction time (min)	COD (mg l^−1^)
1	2	2.46	0.10	30	367.29
2	2	4.92	0.25	60	348.30
3	2	7.38	0.5	90	173.76
4	2	10.26	0.75	120	78.70
5	3	2.46	0.25	90	206.38
6	3	4.92	0.10	120	203.01
7	3	7.38	0.75	30	145.04
8	3	10.26	0.5	60	406.02
9	4	2.46	0.5	120	289.10
10	4	4.92	0.75	90	213.59
11	4	7.38	0.10	60	255.45
12	4	10.26	0.25	30	156.59
13	5	2.46	0.75	60	210.71
14	5	4.92	0.5	30	360.59
15	5	7.38	0.25	120	145.76
16	5	10.26	0.10	90	53.40
*K*1	242.01	268.37	219.79	257.38	
*K*2	240.11	281.37	214.26	305.12	
*K*3	228.68	180.00	307.37	161.78	
*K*4	192.62	173.68	162.01	179.14	
*R*	49.40	107.70	145.36	143.34	

Test results of the L16 (4^4^) orthogonal experiment are presented in table 1. As can be seen from table 2, most of the organic compounds were degraded in the treatment of folic acid wastewater under the condition of experiment 16. The optimal condition is that the air sparge rate is 0.75 l min^−1^, reaction time is 90 min, current density 10.26 mA cm^−2^ and pH is 5. Nidheesh *et al.* [15] found that the optimum pH and Fe^2+^ concentration for 10 mg l^−1^ RhB solution was found to be 3 mg l^−1^ and 10 mg l^−1^, respectively. It may be that the different components of the treated wastewater result in inconsistent reaction conditions.

**Table 2.** Changes with time at different concentrations of folic acid wastewater. Notes: initial pH 5; air sparging rate 0.75 l min^−1^; current density 10.26 mA cm^−2^; [Fe^2+^] 1.0 mM; reaction time 90 min.

time (min)	5	10	15	20	30	45	60	90
*c*_0_ = 1100 mg l^−1^	1056.71	1040.38	1003.29	881.42	693.10	629.87	549.66	441.38
*c*_0_ = 800 mg l^−1^	595.05	925.47	543.00	598.04	491.84	449.48	342.89	277.81
*c*_0_ = 500 mg l^−1^	506.30	508.07	485.87	474.04	436.16	393.12	312.83	198.12
*c*_0_ = 200 mg l^−1^	265.69	254.91	247.75	230.22	209.22	170.04	149.21	117.84

The *K* of pH, current density, air sparge rate and reaction time are listed in table 1; at the same time, the *R* of pH, current density, air sparge rate and reaction time were 49.40, 107.70, 145.36 and 143.34, respectively. The *K* is the average of a certain factor and level and can be used to determine the global optimal extraction condition; *R* is the range of *K*, which can show the effect of a certain factor on the removal efficiency.

### 3.3 Study on degradation products of folic acid

**Figure 3.**
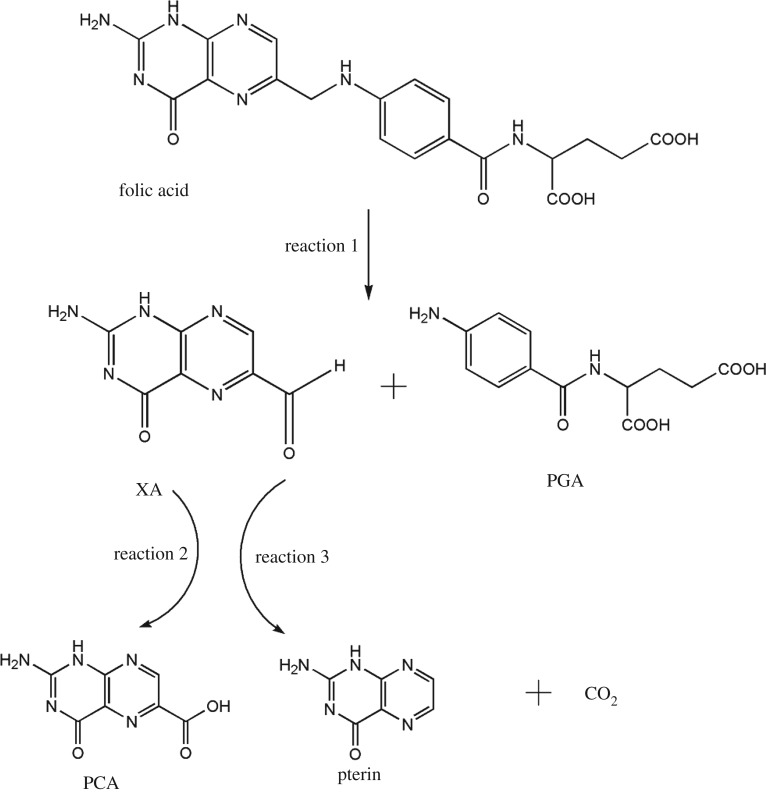
Degradation pathway of folic acid.

To study the degradation pathway of folic acid, LC-MS analysis of the treatment wastewater obtained by experiment 16 was conducted. The LC-MS showed that four degradation products were clearly identified, as shown in figure 3, and this was consistent with the literature [37,38]. The degradation path of folic acid is shown in the electronic supplementary material, figure S1; the electro-Fenton method led to an initial folic acid decomposition into *p*-aminobenzoyl-glutamic acid (PGA) and xanthopterin (XA), just like reaction 1 in figure 3. Oxidation and reduction reactions occurred during electro-Fenton treatment, resulting in different sub-products. As we can see from reaction 2 and reaction 3 in figure 3, XA was oxidized to pterine-6-carboxylic acid (PCA) and XA can be converted into pterin and carbon dioxide (CO_2_), and CO_2_ overflowed from the solution. From the whole process, we know that a part of the large folic acid molecules is converted into carbon dioxide, which is removed from the wastewater, and a part converted into small molecules, thereby reducing the COD in wastewater.

### 3.4. Kinetics of degradation of folic acid wastewater

#### 3.4.1. Order of kinetics of degradation of folic acid wastewater

To make the chemical reaction apply to the production and achieve the ideal reaction speed, we have studied the reaction kinetics. Under the conditions that the air sparge rate is 0.75 l min^−1^, reaction time is 90 min, current density is 10.26 mA cm^−2^and pH is 5, folic acid wastewater with a COD concentration of 1100 mg l^−1^ reacted for 90 min; the concentration of folic acid was measured at each interval of time to establish [*C*] − *t* fitted coordinates (zero order), ln[*C*] − *t* fitted coordinates (first order) and 1/[*C*] − *t* fitted coordinates (second order) [39,40].

**Figure 4.**
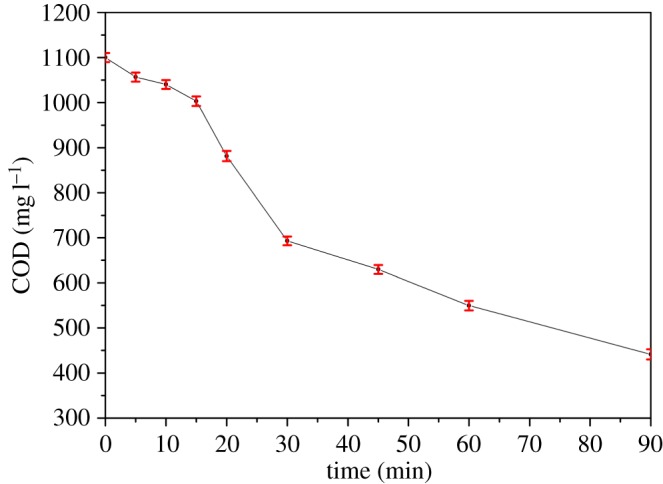
Concentration of folic acid wastewater changes with time. *Conditions*: *c*_0_ = 1100 mg l^−1^; initial pH 5; air sparging rate 0.75 l min^−1^; current density 10.26 mA cm^−2^; [Fe^2+^] 1.0 mM; reaction time 90 min.

As can be seen from figure 4, with the passage of time, the concentration of folic acid in the solution was gradually decreasing, which indicated that folic acid was continuously degraded. To better understand the reaction process, we studied the kinetics of folic acid degradation. The kinetics of reaction involves study of the effect of the chemical reaction rate and various factors influencing the chemical reaction rate.

**Figure 5.**
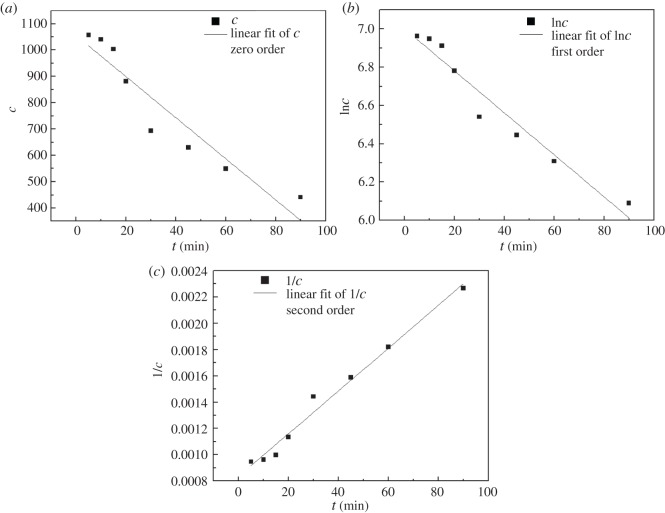
Kinetic fitting of folic acid wastewater. Conditions: initial pH 5; air sparging rate 0.75 l min^−1^; current density 10.26 mA cm^−2^; [Fe^2+^] 1.0 mM; reaction time 90 min.

Zero-order, first-order and second-order linear fitting results obtained are shown in figure 5. By comparison, it is found that the *R*^2^ is 0.98123 when the reaction is fitted to second order, so it can be concluded that the degradation of folic acid is close to a second-order reaction due to the constant concentration of hydroxyl radicals, and the second-order reaction is given by

3.1 $$\frac{1}{c}-\frac{\text{1}}{\text{1100}}=1.627E-5t.$$

#### 3.4.2. Effects of folic acid concentration on degradation kinetic constants

Under the conditions that the air sparging rate is 0.75 l min^−1^, reaction time is 90 min, current density is 10.26 mA cm^−2^ and pH is 5, the concentrations of simulated folic acid wastewater are 1100 mg l^−1^, 800 mg l^−1^, 500 mg l^−1^ and 200 mg l^−1^, respectively. The concentration of folic acid was measured at each interval of time and the results are given in table 2.

As can be seen from table 2, with the passage of time, the concentration of folic acid in the solution was gradually reduced. The data in table 2 for zero-order, first-order and second-order linear fitting are shown in figure 5. The *R*^2^ values of the obtained second order are 0.98123, 0.97064, 0.98063 and 0.99331, respectively. It was proved that the reaction of folic acid degradation was a second-order reaction.

**Figure 6.**
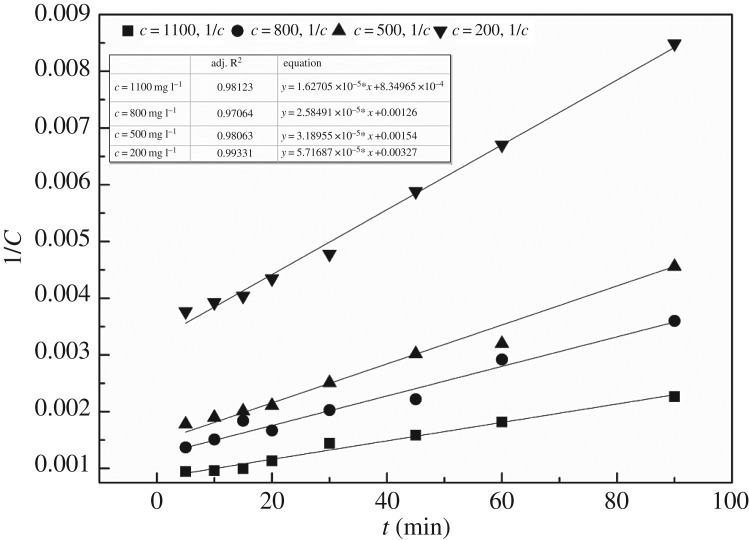
Fit diagram of second order for degradation of different concentrations of folic acid. Conditions: initial pH 5; air sparging rate 0.75 l min^−1^; current density 10.26 mA cm^−2^; [Fe^2+^] 1.0 mM; reaction time 90 min.

It can be seen from figure 6, with the increase of the initial concentration of folic acid from 200 mg l^−1^ to 1100 mg l^−1^, the slope of the equation decreased, which indicated that the 1/*c* increased more rapidly; that is to say, the lower the folic acid concentration, the faster is the degradation of folic acid. We can make the conclusion that when the initial concentration of folic acid was low, it was beneficial to the degradation reaction of folic acid.

### 3.5. The circulation of powdered activated carbon

**Figure 7.**
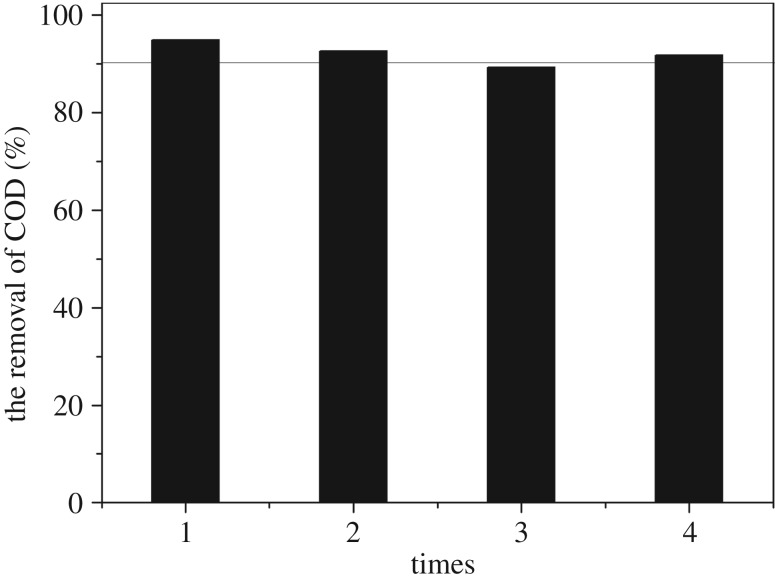
Experiments on powdered activated carbon cycle. Conditions: initial pH 5; air sparging rate 0.75 l min^−1^; current density 10.26 mA cm^−2^; [Fe^2+^] 1.0 mM; reaction time 90 min.

To achieve the purpose of recovery of PAC, we used the PAC after the reaction and carried out a series of cycle experiments. The results are shown in figure 7. As can been seen from the figure, with the first use of PAC, COD removal can reach 94.87%; in the second cycle, the COD removal can still reach more than 90%; only the removal rate in the third cycle is slightly lower, close to 90%. Overall, the cyclic use of PAC is better, presumably because the PAC is only the third pole of the conductive material in the sample, not the adsorbent.

## 4. Conclusion

This study has demonstrated that the electric-Fenton method using a three-dimensional electrode is an efficient method for the degradation of folic acid. The reaction process takes place without adding H_2_O_2_ and only needing one addition of Fe^2+^; then Fe^2+^ can be obtained by Fe^3+^ and this reduces the formation of iron mud. The reaction conditions were optimized and it can be found that when the air sparging rate is 0.75 l min^−1^, current density is 10.26 mA cm^−2^, pH is 5 and the reaction time is 90 min, the COD removal rate can reach 94.87%. This also indicated that the electric-Fenton method using the three-dimensional electrode had a great influence on COD removal.

Throughout the reaction, folic acid decomposed into PGA and XA; part of the XA was oxidized to PCA and the remaining XA also can be converted into pterin and CO_2_ by the electro-Fenton method. Under the optimum conditions, kinetic analysis was carried out and it can be concluded that the degradation of folic acid is a second-order reaction. The kinetics of degradation of folic acid wastewater provided a deep insight into the whole reaction process. In addition, how to improve the utilization rate of electric energy and thus save energy is also a new research direction.

## Supplementary Material

Cover letter

## Supplementary Material

Supplementary materials

## Supplementary Material

Supporting data

## Data accessibility

The datasets supporting this article have been uploaded as part of the electronic supplementary material.
