# Supplementary material for: Degradation of folic acid wastewater by electro-Fenton with three-dimensional electrode and its kinetic study
Source: R Soc Open Sci. 2018 Jan 17;5(1):170926. doi: 10.1098/rsos.170926 (PMC5792884; doi:10.1098/rsos.170926)
Supplement: Supplementary materials [file rsos170926supp2.doc]

**Supplementary materials:**

**Table 1** Characteristics of PAC

**Table 1** Characteristics of PAC

| Sample | Particle size（mesh） | specific surface area(m^2^/g) | Water (%) | Iodine value (mg/g) | methylene blue (mg/g) |
| --- | --- | --- | --- | --- | --- |
| PAC | 150 | 400 | ≤5 | 1000 | 150 |

**Fig. 1.** LC-MS of degradation pathway of folic acid


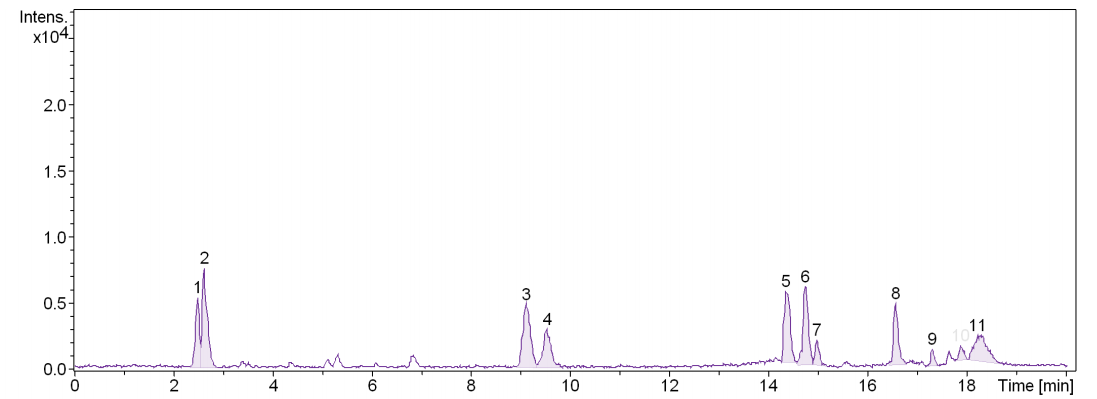

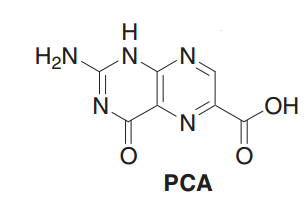

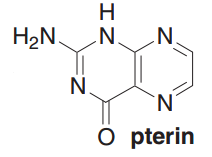

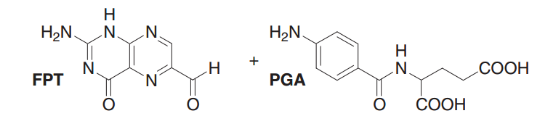


**Fig. 1.** LC-MS of degradation pathway of folic acid
