# Supplementary material for: Degradation of folic acid wastewater by electro-Fenton with three-dimensional electrode and its kinetic study
Source: R Soc Open Sci. 2018 Jan 17;5(1):170926. doi: 10.1098/rsos.170926 (PMC5792884; doi:10.1098/rsos.170926)
Supplement: Cover letter [file rsos170926supp1.doc]

Dear Editor,

Thank you and the reviewers’s for their valuable comments. We have revised the manuscript (Manuscript ID: RSOS-170926) and sent the revised manuscript.

We have sent you the following files: “Responses to reviewers comments”, “a marked-manuscript”and “revised manuscript”. The reply to both reviewer’s and editorial’s comments are listed below. The comments of first, second and editorial are highlighted as red, blue and green, respectively.

Corresponding to:Xu Jin,School of Management, Tianjin University of Technology, Tianjin, 300384, China E-mail: [xujinbox@163.com](mailto:xujinbox@163.com)

Thank you for your consideration.

We are looking forward to hearing from you.

Yours Sincerely

Xu Jin
